# Trajectories of sickness absence among young people with prior depression/anxiety symptoms

**DOI:** 10.1093/eurpub/ckac131.473

**Published:** 2022-10-25

**Authors:** I Alaie, P Svedberg, A Ropponen, J Narusyte

**Affiliations:** 1 Department of Clinical Neuroscience, Karolinska Institutet, Stockholm, Sweden; 2 Finnish Institute of Occupational Health, Helsinki, Finland; 3 Center for Epidemiology & Community Medicine, Stockholm County Council, Stockholm, Sweden

## Abstract

**Background:**

Depression and anxiety are associated with elevated risks of sickness absence (SA), but less is known about the development of SA over time in young people experiencing these mental health conditions. This study aimed to identify latent trajectories of SA in young people with a history of depression and/or anxiety symptoms, while accounting for sociodemographic factors.

**Methods:**

This was an observational cohort study of 1,445 twin individuals who had elevated depression/anxiety symptoms in late adolescence or young adulthood (age range: 19-30), as assessed in Swedish surveys completed in 2005. Through linkage to the national registries, the individuals were prospectively followed from 2006 to 2018. The outcome included consecutive annual net days of SA, which were analyzed using group-based trajectory modeling with zero-inflated Poisson regression. Multinomial logistic regression estimating odds ratios (OR) with 95% confidence intervals (CI) was used to examine the influence of age, sex, and educational level on the resulting trajectory groups.

**Results:**

Four distinct trajectories of SA were identified: ‘high-increasing’ (6%), ‘low-increasing’ (12%), ‘high-decreasing’ (13%), and ‘constant-low’ (69%). The constant-low was used as the reference in all analyses. Increasing age was found to be associated with higher odds of belonging to the low-increasing trajectory (OR = 1.07, 95% CI = 1.02-1.12). Women had higher odds of belonging to the low-increasing trajectory (OR = 1.67, 95% CI = 1.10-2.53), compared to men. Higher education was associated with lower odds of belonging to the high-increasing (OR = 0.34, 95% CI = 0.22-0.54) and high-decreasing (OR = 0.59, 95% CI = 0.43-0.81) trajectories, compared to lower education.

**Conclusions:**

Distinct group-based trajectories of SA were identified in young people with early depression/anxiety symptoms. Targeted and timely public health strategies aiming to improve adolescent and young adult mental health may help reduce SA in the long run.

**Key messages:**

• We identified four trajectories of sickness absence in young people with common mental health problems.

• Public health efforts to improve mental health may reduce sickness absence in vulnerable groups.

